# Effects of different load weights on the work performance and physiological and hematobiochemical responses in working water buffalo

**DOI:** 10.14202/vetworld.2023.2349-2357

**Published:** 2023-11-27

**Authors:** Visoky Paján-Jiménez, Fernando David Pazmiño-Rodríguez, Patricia Roldán-Santiago, Anna Dutro-Aceves, Luis Alberto de la Cruz-Cruz, Cristian Larrondo

**Affiliations:** 1. Maestría en Producción Animal, Universidad Tecnológica Equinoccial, Santo Domingo de los Tsachilas, Ecuador; 2 Universidad Técnica del Norte, Ecuador, Facultad de Ingeniería en Ciencias Agropecuarias y Ambientales, Carrera de Ingeniería Forestal; 3 Departamento de Reproducción, Facultad de Medicina Veterinaria y Zootecnia, Universidad Nacional Autónoma de México, Av. Universidad, Colonia, C.U. C.P. 04510 Ciudad de México, México; 4 Escuela de Ciencias de la Salud, Medicina Veterinaria y Zootecnia, Universidad del Valle de México-Coyoacán, Calzada de Tlalpan 04910, Ciudad de México, México; 5 Preservación del Bienestar Animal/Manejo de la Fauna Silvestre, Departamento de Producción Agrícola y Animal, Universidad Autónoma Metropolitana, Xochimilco, Calzada del Hueso 1100, Col. Villa Quietud, Ciudad de México, 04960, México; 6 Núcleo de Investigaciones Aplicadas en Ciencias Veterinarias y Agronómicas, Facultad de Medicina Veterinaria y Agronomía, Universidad de Las Américas, Viña del Mar, Chile; 7 AWEC Advisors S.L. Parc de Recerca Universitat Autònoma de Barcelona, Cerdanyola del Vallès, España

**Keywords:** biochemical responses, draught buffalo, hematological responses, physiological responses

## Abstract

**Background and Aim:**

Working animals are important in agriculture because they play a role in various agricultural activities, including milk and meat production. Thus, they contribute to the development of rural communities. In this study, we aimed to evaluate the effects of different load weights on the work performance and the physiological and hematological responses of working water buffalo (*Bubalus bubalis*).

**Materials and Methods:**

The work performances of 12 buffaloes (average weight 782.16 ± 21.62 kg) transporting 200, 350, and 500 kg of African palm fruits in metal baskets placed on their backs were evaluated. Work performance variables evaluated immediately after work were as follows: total number of trips (n), total weight (kg), distance traveled (km/day), working period (h), stopped time (h), and average speed (km/h). In addition, we evaluated physiological, biochemical, and hematological variables at three different times: before the start of work, immediately after work, and on the rest day.

**Results:**

Among the load weights, 500 kg (total load carried = 4,138.88 kg) improved work efficiency compared to 200 kg loads (total load = 3,322.22 kg) (p = 0.0281). However, 500 kg loads resulted in slower average speed (2.4 km, p = 0.0164), shorter working period (2.39 h, p < 0.0001) and distance traveled (7.29 km, p < 0.0001), and less total number of trips (8.27 trips, p < 0.0001) compared to 350 and 200 kg load weights (3.45 and 3.52 km/h, 2.55 and 2.79 h, 8.71 and 9.75 km, 10.94 and 16.61 trips, respectively); and the heaviest loads resulted in significantly higher (p < 0.005) respiratory rate, pulse, heart rate, rectal temperature, glucose, lactate dehydrogenase, creatine kinase, total protein, white blood cell count, neutrophils, lymphocytes, monocytes, eosinophils, and basophils. In contrast, was associated with lower levels of red blood cells, hemoglobin, and hematocrit compared to lower loads. All differences were more pronounced in 500 kg (p < 0.005) compared to 200 kg loads.

**Conclusion:**

Working buffaloes responded to work related to the transport of African palm fruits through various physiological, biochemical, and hematological changes. However, some variables remained close to the reference values reported in the literature for water buffaloes, and in general, all variables were reestablished during the rest day, indicating that these animals have adapted to working conditions.

## Introduction

The buffalo (*Bubalus bubalis*) was domesticated around 3000–6000 years ago and since then, it has been an important livestock animal used as both a food source and as a draught animal in tropical and subtropical regions [1, 2]. Buffaloes were introduced at the end of the 19^th^ century into the continent of America, where they were first utilized as a draught animal, then later on for meat production, and finally for dairy [3, 4]. In Ecuador, male buffaloes work as draught animals in African palm (*Elaeis guineensis)* plantations, where they are mainly involved in harvesting the ripe fruit and transporting them in containers. Buffaloes transport the fruits to the processing plants that extract the oil used for human consumption [5, 6].

Draught animals are an important resource in agriculture and urban transport, mainly in developing countries, because they tend to contribute more to economic and social development in these countries [7]. Despite motorization, working animals play an important role for small and medium producers, especially in sectors where it is impossible to operate machinery due to irregular topography or to avoid soil compaction [8–11]. Working animals are used for a variety of purposes, such as drawing agricultural implements, hauling animal-drawn carts, providing motive power to devices, and carrying loads on their backs as pack animals [12]. Buffaloes are long-lived, adapt well to the environment, are easily domesticated, and are able to pull twice the load as that drawn by cattle of the same weight, size, and age [11, 13]. In addition, buffaloes are an integral part of sustainable agriculture because of their capacity to produce milk, meat, and manure [14].

The health status of animals or the functions of a range of body systems are typically determined based on the evaluation of physiological variables, hemograms, and plasma or serum biochemistry [15–17]. Such evaluations are also performed in response to various conditions, such as physical exercise [18–20]. Changes in physiological parameters and the different components of plasma biochemistry of animals reflect the stress of work imposed on working animals; these have been used to evaluate bullocks [21–24], donkeys [25, 26], horses [20, 27], mules [28, 29], dromedary camels [30, 31], and dogs [32, 33].

Few experimental studies have investigated work performed by buffaloes [34–36]. Moreover, comprehensive information on work performance and the physiological, hematological, and biochemical changes that occur in response to work in buffaloes under field conditions is not available.

Therefore, this study aimed to evaluate the effects of different load weights on work performance and the physiological and hematological responses of working water buffalo (*B. bubalis*) used to transport African palm fruits.

## Materials and Methods

### Ethical approval

This study was approved by the General Direction Committee of postgraduate courses of the Equinoctial Technological University and the experimental procedures were carried out in relation to the Organic Law of Agricultural Health (No. 919), Ecuador.

### Study period and location

This study was conducted from August to September 2014 on a commercial farm dedicated to producing oil palm fruit bunches (*E. guineensis* Jacq.) in the province of Esmeraldas, canton Quinindé, Ecuador. The study was conducted in the sixth zone at a height of 165 m above sea level, N 0018 27.9, W 079 17 30.8. During the study, the area had an average temperature of 24.4°C (minimum: 24.00, maximum. 32.40°C), solar radiation of 241.3, and a humidity of 91.7%.

### Animals

Twelve mature (between 7 and 10 years old) male water buffaloes (*B. bubalis*) of the Murrah breed were used in this study. The animals had an average weight of 782.16 ± 21.62 kg and had been doing the same work in the production unit for about 5 years. Their training started at 1 year of age, and they had been trained to pull carts and to carry weight on their backs by for 3 months before starting their usual work. Based on the scale (1–5) developed by Singh *et al*. [37], the buffaloes’ body condition score was between 3 and 4.

**Figure-1:**
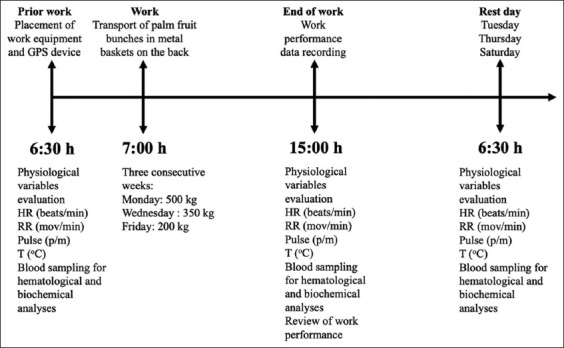
Chronology of the evaluation of work performance variables, physiological, biochemical, and hematological variables in working buffaloes.

The animals were used to transport African palm fruits packed into two separate metal baskets placed on their backs, which is the routine practice in accordance with the following: the animals worked from 7:00 am to 3:00 pm on specified days, during which time the animals transported decreasing weight loads as follows: Monday, 500 kg; Wednesday, 350 kg; and Friday, 200 kg; Tuesday, Thursday, Saturday, and Sunday were rest days. We evaluated all variables according to Figure-1, and we performed these evaluations during three consecutive weeks with the same sequence, thus generating three data points for each variable measured. These data points were averaged for statistical analysis. When the buffaloes were not working, they were placed in a pasture with available shade, water, and forage.

### Work performance

The average speed (km/h) and distance traveled (km) of each buffalo were measured with a global positioning system (GPS) (eTrex Legend^®^ HCx, Garmin International Inc., Kansas, USA) that was placed on the work equipment of each animal during the working day. The data were transferred to the Garmin basecamp program where the information was recorded and stored daily. The number of trips that each animal made to the fruit storage site during the working day was recorded. To calculate the load transported per animal for each trip, the palm fruits were weighed using a portable scale with a capacity of 100 kg (Model NTA, Camry^®^, Guangdong, China), then the weight of the load was multiplied by the number of journeys made by each animal.

### Physiological variables

We measured the following physiological variables: Heart rate (HR), pulse, respiratory rate (RR), and rectal temperature (RT). Heart rate was measured as the number of beats per minute using a stethoscope (Littmann^®^ Lightweight II SE, model 2,450, Massachusetts, USA) placed between the third and fifth intercostal space. The RR was measured by counting the number of movements per minute (mov/min) at the thoracic-abdominal area. The pulse was determined by palpation of the middle coccygeal artery (p/m). Rectal temperature was measured by inserting a flexible clinical thermometer (Microlife^®^, model MT200; interval 32°C–43.9°C ± 0.1°C precision, China) into the rectum of the animal for at least 1 min. The physiological parameters of each animal were based on measurements performed 3 times and subsequently averaged.

### Blood sampling

Blood samples were collected at 30 min before work (6:30 h), immediately after completion of work (15:00 h), and on the rest day (6:30 h) (Figure-1). Before sampling, a local anesthetic (Lidocaine 5%) was applied at the puncture site using an atomizer. All blood samples were aseptically collected by jugular venipuncture into Vacutainer^®^ (Plymouth, United Kingdom) tubes and were divided into two portions. The first portion (4 mL) was collected into anticoagulant ethylenediaminetetraacetic acid (EDTA) tubes and was used to measure hematological parameters. The second portion (6 mL) was collected without anticoagulants and was used for analyzing the biochemical parameters of the serum. All samples were refrigerated (0°C–4°C) and transported to a private laboratory within 2 h after collection, and all hematological and biochemical analyses were performed within 4 h of sample collection.

### Hematobiochemical variables

Twenty microliters of blood samples in EDTA tubes were processed in an automatic hematology analyzer BC-5300 (Mindray^®^ Bio-Medical Electronics Co., Ltd, Shenzhen, China) to determine hematological variables. The following analytes were measured: Red blood cell count (RBC), hemoglobin (Hb), hematocrit (HCT), main corpuscular volume (MCV), mean corpuscular Hb concentration (MCH), RBC distribution (RDW), white blood cells count (WBC), neutrophils (N), lymphocytes (L), monocytes (M), eosinophils (E), and basophils (B).

Blood samples were centrifuged at 2000× *g* for 10 min in plain tubes and the serum was harvested and transferred to Eppendorf tubes. Samples with lipemia and hemolysis were excluded. The following serum components were measured using commercial kits (Doles Reag. Equip. Lab. Ltda, Goiânia, Brazil) and an automated biochemical analyzer BS-120 (Mindray^®^ Bio-Medical Electronics Co., Ltd, Shenzhen, China): Glucose (glu), lactate dehydrogenase (LDH) and creatine kinase (CK) and Total proteins (TP). Quality control provided with the kits was used to verify the performance of the measurement procedures.

### Statistical analysis

All statistical analyses were performed using JMP^®^ version 16 software (SAS Institute Inc., Cary, NC, USA). The data are presented as means ± standard error of the mean. A Shapiro–Wilk test was used to assess the normality of the distribution. Data with a non-normal distribution were log-transformed. All variables were compared with a mixed model for repeated measures, considering the factors time (before and after work and rest day), load weight (200, 350, and 500 kg), and time × load weight interactions as fixed effects; and the animal in each group as a random effect. A mixed model for repeated measures was applied to analyze work performance, considering the load weight as a fixed effect and the animal in each group as a random effect. In both cases, *post hoc* comparisons were performed by applying the Tukey test with a probability level of p < 0.05.

## Results

Buffaloes changed their work performance according to different loading weights, which was reflected in alterations in the physiological variables.

The results of work performance analysis (Table-1) show that the buffaloes working with heavier loads had less total number of trips (p < 0.0001), distance traveled (km) (p < 0.0001), working period (h) (p < 0.0001), and average speed (km/h); while their total weight transported (p = 0.0281) and time stopped (p < 0.0001) values were lower compared to buffaloes with 350 and 200 kg load weights.

**Table-1:** Effect of different weight loads on work performance in working buffaloes.

Variable	Load weight	Value	SEM	p-value
Total number of trips	200 kg	16.61^a^	0.026	<0.0001
	350 kg	10.94^b^	0.56	
	500 kg	8.27^c^	0.58	
Total weight (Kg)	200 kg	3322.22^b^	52.43	0.0281
	350 kg	3830.55^a,b^	197.33	
	500 kg	4138.88^a^	293.61	
Distance traveled (km)	200 kg	9.75^a^	0.13	<0.0001
	350 kg	8.71^b^	0.12	
	500 kg	7.29^c^	0.09	
Working period (h)	200 kg	2.79^a^	0.02	<0.0001
	350 kg	2.55^b^	0.03	
	500 kg	2.39^c^	0.01	
Stopped time (h)	200 kg	5.20^c^	0.01	<0.0001
	350 kg	5.44^b^	0.03	
	500 kg	5.59^a^	0.01	
Average speed (km/h)	200 kg	3.52^a^	0.03	0.0164
	350 kg	3.45^b^	0.02	
	500 kg	3.40^c^	0.03	

^a,b,c^Different letters in the same column indicate significant differences (p < 0.05). SEM=Standard error of the mean

Results of analyses of physiological variables (Table-2) show significant effects of time (p < 0.001), load (p < 0.001), and T × LW interaction (p < 0.01). The passage of time resulted in an increase in all the variables after work, regardless of the load weight (p < 0.05). Among the load weights, 500 kg resulted in more physiological changes after work compared to 200 and 350 kg (p < 0.05).

**Table-2:** Effect of different weight loads and time on physiological variables in working buffaloes.

Response variable	Time	Load weight			SEM	Time	Load weight	Time *Load weight

		200 kg	350 kg	500 kg				
RR (mov/min)	Before	20.00^b,C^	20.00^b,C^	20.00^b,C^				
	After	52.22^a,B^	56.00^a,B^	62.55^a,A^	0.44	<0.001	<0.001	<0.001
	Rest	20.44^b,C^	20.88^b,C^	20.88^b,C^				
Pulse (pulse/min)	Before	37.11^b,C^	37.11^b,C^	36.88^b,C^				
	After	59.55^a,B^	61.44^ª,B^	70.11^ª,A^	0.55	<0.001	<0.001	<0.001
	Rest	37.11^b,C^	37.11^b,C^	36.88^b,C^				
HR (beats/min)	Before	43.55^b,C^	44.00^b,C^	44.00^b.C^				
	After	63.77^a,B^	65.22^a,B^	73.55^a,A^	0.39	<0.001	<0.001	<0.001
	Rest	45.00^b,C^	44.88^b,C^	44.72^b,C^				
RT (°C)	Before	38.20^b,D^	38.20^b,D^	38.20^b,D^				
	After	39.63^a,C^	39.79^ª,B^	40.16^ª,A^	0.02	<0.0001	<0.0001	0.0024
	Rest	38.20^b,D^	38.20^b,D^	38.21^b,D^				

^a,b,c^Different letters in the same column indicate significant differences due to the effect of time within each load weight (p < 0.05). ^A,B,C,^DDifferent letters in the same row indicate significant differences due to time effect of load weight regardless of evaluation time (p < 0.05). RR=Respiratory rate, HR=Heart rate, RT=Rectal temperature, SEM=Standard error of the mean

Table-3 shows the effects of time and loading weight on biochemical variables of buffaloes working with different load weights. The effects of time were significant (p < 0.001) for all variables. Significant effects of load weight were observed only for glu (p < 0.001) and TP (p = 0.0005). Likewise, T × LW interactions were significant for glu (p < 0.001), LDH (p < 0.001), and TP (p < 0.0001). All the biochemical variables evaluated in buffaloes increased their values immediately after work started, regardless of the load weight. Meanwhile, the highest glu and TP values were observed after work among the buffaloes carrying a load of 500 kg compared to those loaded with 200 kg.

**Table-3:** Effect of different weight loads and time on biochemical variables in working buffaloes.

Response variable	Time	Load weight			SEM	Time	Load weight	Time *Load weight

		200 kg	350 kg	500 kg				
Glucose (mg/dL)	Before	50.27^b,CD^	46.88^b,D^	53.77^b,C^				
	After	67.66^ª,B^	67.66ª^,B^	104.66^ª,A^	1.51	<0.001	<0.001	<0.001
	Rest	50.25^b,CD^	46.75^b,D^	53.88^b,C^				
LDH (UI/L)	Before	11.25^b,A^	9.83^b,A^	8.65^b,A^				
	After	16.67ª^,A^	20.81^ª,A^	22.51^ª,A^	0.27	<0.001	0.2159	<0.001
	Rest	11.26^b,A^	9.78^b,A^	8.61^b,A^				
CK (g/dL)	Before	9.79^b,A^	19.14^b,A^	13.47^b,A^				
	After	196.50^a,A^	221.07^ª,A^	231.52^ª,A^	29.50	<0.001	0.7384	0.9740
	Rest	9.85^b,A^	19.12^b,A^	13.48^b,A^				
TP (g/dL)	Before	6.26^b,C^	5.73^b,D^	6.03^b,CD^				
	After	6.82ª^,B^	7.05ª^,AB^	7.44ª^,A^	0.09	<0.0001	0.0005	<0.0001
	Rest	6.27^b,C^	5.74^b,D^	5.96^b,CD^				

^a,b,c^Different letters in the same column indicate significant differences due to the effect of time within each load weight (p < 0.05). ^A,B,C,D^Different letters in the same row indicate significant differences due to time effect of load weight regardless of evaluation time (p < 0.05). LDH=Lactate Dehydrogenase, CK=Creatine kinase, TP=Total protein, SEM=Standard error of the mean

Table-4 shows the changes in RBCs of the buffaloes according to time and load weight. All variables except RDW were significantly affected by time (p < 0.0001), load weight (p < 0.001) and T × LW interactions (p < 0.05). All variables decreased immediately after work compared to their values before work and during the rest day (p < 0.0001), although these changes were only observed in MCV (fL) and in MCH (pg) when the load was 500 kg (p < 0.0001). The highest values of RBC (×10^6^/μL), Hb (g/dL), and HTC (%) were observed after work, particularly when the load was 500 kg (p < 0.05). Similarly, the lowest values of MCV (fL) and MCH (pg) were observed with the 500 kg load.

**Table-4:** Effect of different weight loads and time on red cells in working buffaloes.

Response variable	Time	Load weight			SEM	Time	Load weight	Time *Load weight

		200 kg	350 kg	500 kg				
RBC (×10^6^/µL)	Before	9.86^ª,A^	9.57^ª,A^	9.93^ª,A^				
	After	7.09^b,C^	6.52^b,C^	8.90^b,B^	0.17	<0.0001	<0.0001	<0.0001
	Rest	9.81^ª,A^	9.54^ª,A^	9.88^ª,A^				
Hb (g/dL)	Before	16.33^ª,A^	16.72^ª,A^	17.02^ª,A^				
	After	11.93^b,C^	11.43^b,C^	13.62^b,B^	0.35	<0.0001	0.0001	0.0227
	Rest	16.31^ª,A^	16.51^ª,A^	16.93^ª,A^				
HCT (%)	Before	53.61^ª,A^	54.55^ª,A^	55.82^ª,A^				
	After	39.07^b,C^	37.17^b,C^	44.29^b,B^	1.16	<0.0001	0.0002	0.0418
	Rest	53.83^ª,A^	54.52^ª,A^	55.91^ª,A^				
MCV (fL)	Before	54.33^ª,C^	56.97^ª,A^	56.20^ª,ABC^				
	After	55.11^ª,ABC^	56.83^ª,AB^	49.59^b,D^	0.42	<0.0001	<0.0001	<0.0001
	Rest	54.84^ª,BC^	57.06^ª,A^	56.58^ª,AB^				
MCH (pg)	Before	16.57^ª,C^	17.48^ª,A^	17.14^ª,ABC^				
	After	16.83^ª,BC^	17.47^ª,A^	15.25^b,D^	0.13	<0.0001	<0.0001	<0.0001
	Rest	16.62^ª,C^	17.29^ª,AB^	17.14^ª,ABC^				
RDW (%)	Before	0.16^ª,A^	0.16^ª,A^	0.16^ª,A^				
	After	0.16^ª,A^	0.17^ª,A^	0.16^ª,A^	0.00	0.0906	0.2608	0.2826
	Rest	0.16^ª,A^	0.16^ª,A^	0.16^ª,A^				

^a,b,c^Different letters in the same column indicate significant differences due to the effect of time within each load weight (p < 0.05). A, B, C, DDifferent letters in the same row indicate significant differences due to time effect of load weight regardless of evaluation time (p < 0.05). RBC=Red blood cells, Hb=Hemoglobin, HCT=Hematocrit, MCV=Mean corpuscular volume, MCH=Mean corpuscular hemoglobin, RDW=Red blood distribution width, SEM=Standard error of the mean

Table-5 shows the effects of time and load weight on WBCs. All variables changed significantly through time (p < 0.001), while significant effects of load weight and T × LW interaction were only observed in lymphocytes (p = 0.0001 and p = 0.0037, respectively), monocytes (p < 0.0001 and p < 0.0001, respectively), eosinophils (p = 0.0015 and p < 0.0001, respectively), and basophils (p = 0.0013 and p < 0.0001, respectively). The values for all WBCs except eosinophils were high when the buffaloes worked with a load of 500 kg, while basophil levels were high when the load was 200 kg (p < 0.001). The effect of load weight varied depending on the evaluation time (p < 0.0001), except in WBC (p = 0.2072) and neutrophil (p = 0.3301) counts, which showed no significant changes (p > 0.05).

**Table-5:** Effect of different weight loads and time on white cells in working buffaloes.

Response variable	Time	Load weight			SEM	Time	Load weight	Time *Load weight

		200 kg	350 kg	500 kg				
WBC count (×10^3^/µL)	Before	8.47^b,A^	7.97^b,A^	7.83^b,A^				
	After	11.03^ª,A^	10.93^ª,A^	11.43^ª,A^	0.26	<0.001	0.2072	0.2297
	Rest	8.41^b,A^	7.97^b,A^	7.82^b,A^				
Neutrophils (×10^3^/µL)	Before	3.56^b,A^	3.79^b,A^	3.61^b,A^				
	After	5.22^ª,A^	5.28^ª,A^	5.68^ª,A^	0.18	<0.001	0.298	0.3301
	Rest	3.56^b,A^	3.82^b,A^	3.60^b,A^				
Lymphocytes (×10^3^/µL)	Before	4.75^b,B^	4.02^b,BC^	4.07^b,C^				
	After	5.17a^,AB^	5.16^ª,AB^	5.44^ª,A^	0.13	<0.0001	0.0001	0.0037
	Rest	4.71^b,B^	4.01^b,C^	4.07^b,C^				
Monocytes (×10^3^/µL)	Before	0.09^b,D^	0.10^b,D^	0.09^b,D^				
	After	0.35^ª,A^	0.26^ª,B^	0.17^ª,C^	0.01	<0.0001	<0.0001	<0.0001
	Rest	0.07^b,D^	0.07^b,D^	0.07^b,D^				
Eosinophils (×10^3^/µL)	Before	0.00^b,B^	0.00^b,B^	0.00^a,B^				
	After	0.21^ª,A^	0.155^ª,A^	0.04^ª,B^	0.01	<0.0001	0.0015	<0.0001
	Rest	0.01^b,B^	0.01^b,B^	0.01^ª,B^				
Basophils (×10^3^/µL)	Before	0.04^ª,B^	0.04^b,B^	0.04^b,B^				
	After	0.05^ª,B^	0.06^ª,A^	0.08^ª,A^	0.00	<0.001	0.0013	<0.0001
	Rest	0.04^ª,B^	0.04^b,B^	0.04^b,B^				

^a,b,c^Different letters in the same column indicate significant differences due to the effect of time within each load weight (p < 0.05). ^A,B,C,D^Different letters in the same row indicate significant differences due to time effect of load weight regardless of evaluation time (p < 0.05). WBC=White blood cells count, SEM=Standard error of the mean

## Discussion

The results of this study indicate that buffaloes carrying heavier loads tend to move slower compared to buffaloes with lower loads. Similar to horses, working animals minimize energy consumption by moving slower during work [38]. In the present study, the average movement speed was 3.45 km/h, which differs from speeds in buffaloes performing different farm works such as ploughing (2.16 to 3.2 km/h) [14, 39, 40] and carting (5.2 km/h) [11]. These speeds are completely dependent on the type of work and the weight of the load. Although large buffalo bulls are able to pull heavy loads, they are as maneuverable as small and compact animals and hence move slowly [40]. In addition, under regular topography, buffaloes tend to move slower than horses, mules, or donkeys, although they are very efficient in muddy or flooded lands [14].

In swamp buffaloes weighing 380 kg, it has been observed that after 3 h of work, displacement speed of 3.81 km/h decreases when the load is 5% of body weight, and when the load weight is increased to 15% of body weight, the speed decreases to 3.02 km/h [41]. We maintained a displacement speed of between 3.40 and 3.52 km/h, regardless of the load weights. However, 200, 350, and 500 kg load weights in this study represented 26%, 45%, and 64% of body weight, respectively. Compared to the younger animals used in other studies [11, 41], the buffaloes in the present study were larger and more experienced, thus they had a greater capacity to carry heavier loads.

We observed that increasing the load weight on buffaloes improved productive efficiency, that is, more kilograms of African palm fruits were transported in fewer trips per day. However, the working time in the present study was shorter (2.57 h on average) compared to that reported by Van Thu [42] in Vietnam, where the buffaloes worked between 5.05 and 5.39 h/day. In another study conducted in India, buffaloes traveled longer distances (72.0 ± 0.57 km/day) during work periods lasting between 8.0 ± 0.05 h and 9.5 ± 0.06 h [11], which are longer than those of the present study (i.e., our buffaloes only traveled between 7.29 and 9.75 km/day during work periods lasting between 2.39 and 2.79 h.

The type, intensity, and duration of work cause transitory physiological and metabolic changes in animals [43]. For example, temperature, respiration, and pulse increase in response to physical work [44]. Muscular activity activates the sympathetic-adrenal medullary axis, which induces the release of epinephrine, leading to the activation of a and b receptors that cause diverse physiological adjustments in response to stressors [45, 46]. The increases in RR, pulse, HR, and RT observed in the present study are consistent with similar responses in Surti buffaloes [47], bullocks of Hariana cattle [24], and equids [20, 48]. The increase in HR during exercise occurs in response to increased metabolic rates due to adrenaline and glycolysis; and also due to an increase in cardiac contraction to maintain an adequate blood flow for perfusion and tissue oxygenation, which is related to the increase in RR and pulse [43, 49]. The change in body temperature is due to heat production resulting from muscle contraction during work [24]. The increases in values of physiological parameters are similar to those observed in working buffaloes after ploughing muddy fields for 1–5 h [44].

The results of previous studies by Mahardika *et al*. [9], Mahardika *et al*. [41], and Vivek *et al*. [48] support the hypothesis that the greater availability of glu at the plasma level increases the ability to perform prolonged or higher-intensity work, and therefore, efficient glu metabolism is an important indicator of better physical endurance. Our results are consistent with this view, as glu levels reached maximum values when the load was 500 kg. The glu values observed in the present study are consistent with those in adult buffaloes previously reported by Mahardika *et al*. [9], Mahardika *et al*. [41], and Kuralkar *et al*. [50]. In general, glu concentrations above reference values can be attributed to the activation of the hepatic processes of glycogenolysis and gluconeogenesis, which is a response to the energetic need to maintain muscle activity. In addition, increases in blood levels of cortisol and catecholamines, concomitant with a reduction in insulin, cause an increase in blood glu [43, 45].

In general, CK levels in the blood are associated with muscle cell damage and disturbance following strenuous exercise that increases muscle enzyme activity [38, 51, 52]. In this study, we observed significantly higher CK values after working, regardless of load weight. However, CK levels are also dependent on the physical performance of the animal, and therefore, maximum plasma CK activity does not provide a realistic assessment of the amount of muscle damage [43]. Previous studies have reported that CK and LDH levels are more related to increased muscle cell permeability during physical work than to actual tissue damage due to heavy workload or mechanical damage [20, 27, 53]. This study showed an increase in TP levels immediately after work. Although TP levels have been used to assess hydration status [54], increased serum protein concentration may also result from a small reduction in plasma volume, rather than real dehydration caused by exercise [27].

The values for RBC, Hb, and HCT that we obtained from buffaloes are slightly higher than the ranges of those reported in buffaloes by Rocha *et al*. [17]; however, our MCV, MCH, leukocytes, neutrophils, lymphocytes, monocytes, eosinophils, and TP values are within the ranges found by Rocha *et al*. [17]. Moreover, our RBC and Hb values are consistent with those reported in other studies in buffalo [9, 55].

According to Patel *et al*. [56], elevated erythrocyte values in adult buffalo are due to the action of androgens in enhancing the growth of erythroid progenitor cells in the presence of erythropoietin, leading to a higher rate of erythropoiesis, which may also be related to the levels of Hb and HCT [50]. Likewise, the higher erythrocyte count in adult buffalo may be due to a high basal metabolic rate, leading to a higher rate of erythropoiesis that raises the erythrocyte count [50].

Certain cardiovascular and hematological adaptations are necessary to guarantee the correct supply of oxygen and blood substrates to active muscles during exercise and the release of metabolites [57]. The increases in levels of RCB and Hb vary according to the intensity of work, which is associated with splenic contraction and a greater oxygen demand after physical exercise or immediately after work [27, 58]. We observed a decrease in HTC values after work regardless of the load weights, which may be due to hemodilution due to excessive water consumption, as previously reported by Pereira *et al*. [59]. The animals had continuous access to water during their work.

After work, we observed increases in the levels of leukocytes, neutrophils, lymphocytes, and monocytes compared to pre-exercise levels and rest values. We attribute these increases to neutrophilia and lymphopenia, as well as the redistribution of neutrophils from the peripheral pool into the blood circulation from splenic contractions [53, 58]. Significant increases in the levels of WBC after exercise confirm a significant post-exercise leukocytosis and the so-called stress leukogram [60].

The changes we observed in hematological variables after performing physical work were consistent with the load weights. However, these changes remained within the physiological ranges as previously reported by Rocha *et al*. [17] in water buffaloes. In addition, buffaloes have the capacity to pull loads more than 6 times their own body weight, although their usual load-carrying capacity is 1.5–2.0 tons, that is, 4 times their body weight [6, 11, 13]. In the present study, the maximum load was approximately 64% of the live weight of the evaluated animals. Which would determine that the animals evaluated have been adapted to the transport of African palm fruits.

## Conclusion

Working buffaloes undergo physiological, biochemical, and hematological changes in response to the work of transporting African palm fruits; these changes varied depending of load weights. In addition, the physiological, biochemical, and hematological changes were greater when the load was 500 kg compared to those at loads of 200 and 350 kg. These physiological changes returned to their initial values during the rest days, and in some cases, the imbalances observed immediately after work remained within the physiological ranges of this species, which indicates that these working buffaloes have been habituated to exercise. Future studies should evaluate the recovery time of the physiological variables in response to physical work. It is also necessary to evaluate the physiological impacts and the state of health of the working buffalo undergoing different agricultural and transport tasks with different equipment to determine the resistance of the species to different types of farm work.

## Authors’ Contributions

VP, FDP, PR, AD, LAC, and CL: Study conception and design. VP and FDP: Material preparation and data collection. PR, AD, LAC, and CL: Performed data analysis and interpretation and drafted and revised the manuscript. All authors have read, reviewed, and approved the final manuscript.
